# Supplementary material for: Scaling behavior of the quantum phase transition from a quantum-anomalous-Hall insulator to an axion insulator
Source: Nat Commun. 2020 Sep 10;11:4532. doi: 10.1038/s41467-020-18312-z (PMC7483742; doi:10.1038/s41467-020-18312-z)
Supplement: Supplementary file 1 — Supplementary Information [file 41467_2020_18312_MOESM1_ESM.pdf]

Supplementary Information

Scaling Behavior of the Quantum Phase Transition from a Quantum-Anomalous-Hall Insulator to an Axion Insulator

Xinyu Wu[1], Di Xiao[2], Chui-Zhen Chen[3], Jian Sun[1], Ling Zhang[2], Moses. H. W. Chan[2], Nitin Samarth[2], X. C. Xie[1,4,5], Xi Lin[1,4,5], and Cui-Zu Chang[2]

[1]International Center for Quantum Materials, Peking University, Beijing 100871, China

[2]Department of Physics, The Pennsylvania State University, University Park, PA 16802, USA

[3]Institute for Advanced Study and School of Physical Science and Technology, Soochow University, Suzhou 215006, China

[4]Beijing Academy of Quantum Information Sciences, Beijing 100193, China

[5]CAS Center for Excellence in Topological Quantum Computation, University of Chinese Academy of Sciences, Beijing 100190, China

*Corresponding authors: xilin@pku.edu.cn (X. L.); cxc955@psu.edu (C. Z. C.)

Supplementary Note 1: Quasi-DC measurement method

The traditional two-terminal AC method for measuring large resistance is limited by the capacitance in the measurement circuit. To achieve lower electron temperature, a number of resistor-capacitor (RC) filters have been installed in our dilution fridges. These RC filters create unavoidable capacitance order of 10 nF in the measurement circuit. This capacitance gives an impedance $Z = \frac{1}{i\omega C} \sim 16$ MΩ for 1 Hz, which is much smaller than the sample resistance in the axion insulator regime.

In order to measure resistance up to the order of GΩ, we used a quasi-DC measurement method in our experiment. A low-frequency square wave ($f = 25.3$ mHz) is applied as the output. The voltage excitation is 600 μV, which is small enough to sidestep the observable Joule heating. Current is amplified by a nF CA5350 preamplifier and then measured by a Keithley 2182A nanovoltmeter (named as DMM hereafter). Since this signal is also a square wave (**Supplementary Fig. 1a**), by subtracting the difference of the square wave shape current, we can eliminate the thermal voltage and calculate the measured resistance as $R = \frac{2V}{I_{upper} - I_{lower}}$. Since the voltage and current are quasi-DC, the low frequency 25.3 mHz gives enough measurement time in each period for DMM to measure the DC signal. As a result, the parasitic capacitance in the measurement circuit does not influence the result. This quasi-DC measurement method requires much longer measurement time than the AC lock-in technique, so it is inconvenient when sweeping magnetic field.

We used the quasi-DC measurement method to measure different numbers (1~5) of 400 MΩ resistors connected in series at room temperature. We found that our quasi-DC measurement circuit can provide the correct resistance values up to ~ 2 GΩ (**Supplementary Fig. 1b)**.

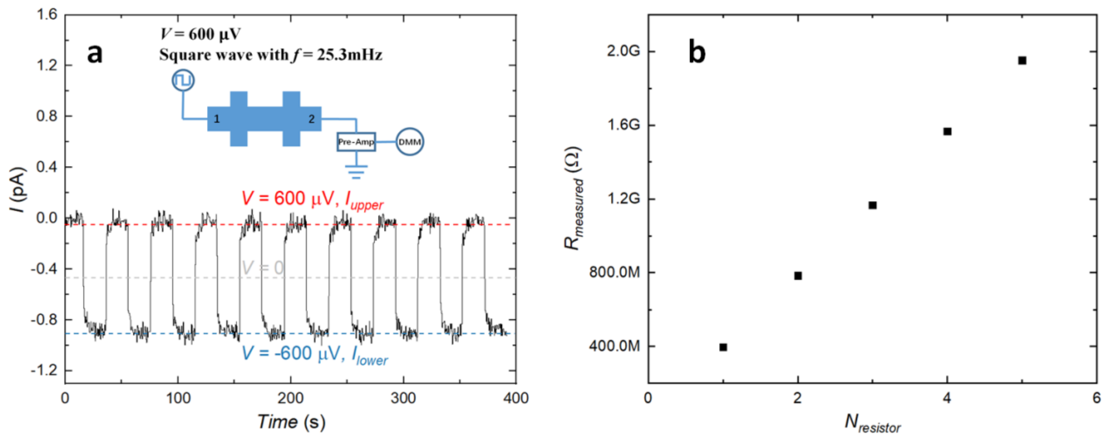

Supplementary Figure 1| Quasi-DC measurement method. (a) Output square wave signal of the quasi-DC measurement. Inset: the quasi-DC simplified measurement circuit. Small excitation of 600 μV with frequency $f = 25.3$ mHz is applied, so DMM has enough time to perform the DC measurement in each period. Due to the zero-point offset and thermal voltage, the output current without excitation is also off from zero. (b) A dummy test at room temperature to check the reliability of the quasi-DC measurement method. The x-axis shows the number of 400 MΩ resistors in series connection, while the y-axis is the measured resistance value using the quasi-DC measurement method.

Supplementary Note 2: Fridge temperature and electron temperature

We trust that the electron temperature is the same as the measured temperature above 45 mK, based on the linear behavior of scaling (**Fig. 3a** and **Supplementary Fig. 2**). When the environmental temperature is 15mK, the electron temperature in our samples is estimated to be ~32 mK assuming the finite size effect doesn't cause saturation at the low-temperature limit.

Our prior works have demonstrated that our dilution refrigerator can cool down electrons below 45 mK [1, 2]. Before acquiring data to determine temperature dependence in our experiment, we carefully checked excitation current dependence at base temperature to make sure our excitation current was still in the linear region. The lower limit of excitation current in a realistic experiment is limited by measurement sensitivity, and the excitation current of 0.3 nA cannot cause self-heating effects in this measurement.

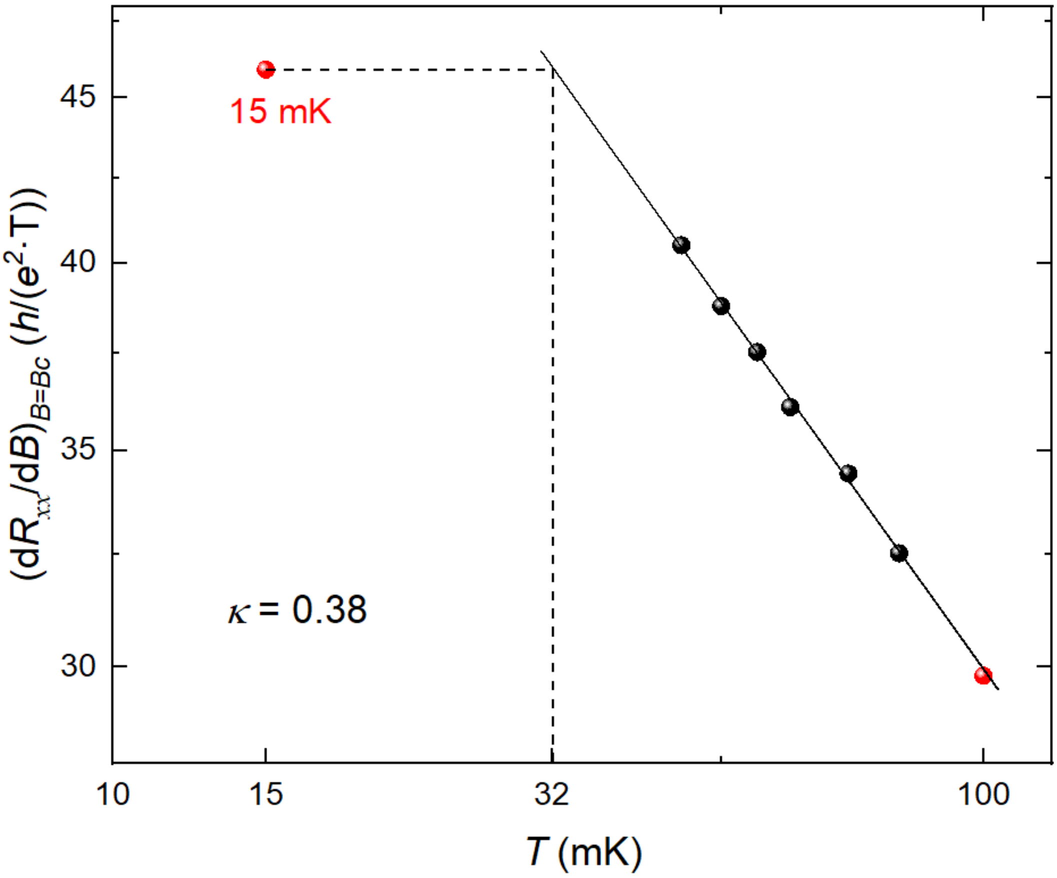

Supplementary Figure 2| Estimation of the electron temperature at 15 mK fridge temperature. The black line is the linear fit of $(\frac{dR_{xx}}{dB})_{B=B_c}$ versus temperature on a log-log scale at temperature range 45 mK ≤ T ≤ 80 mK.

Supplementary Note 3: Scaling behavior analysis based on Hall resistance R_{xy}

We have also analyzed the scaling behavior of the QAH to axion insulator transition based on the R_{xy} data. The critical exponent κ from the R_{xy} data is ~ 0.39, consistent with the scaling analysis based on R_{xx} data described in the main text. **Supplementary Fig. 3a** shows the magnetic field dependence of R_{xy} between 45 mK ≤ T ≤ 100 mK around the crossing point. The crossing point of the R_{xy} data is B_c ~ 0.967 T, slightly different from B_c = 0.979 T for R_{xx}. We speculate that this is likely a result of the influence of mixing from much larger R_{xx}. For instance, R_{xx} is almost twice of R_{xy} at 0.967 T, so the influence of mixing is more pronounced for R_{xy}. Therefore, the linear fit based on R_{xy} is not as good as the R_{xx} fit, as shown in **Supplementary Fig. 3b**.

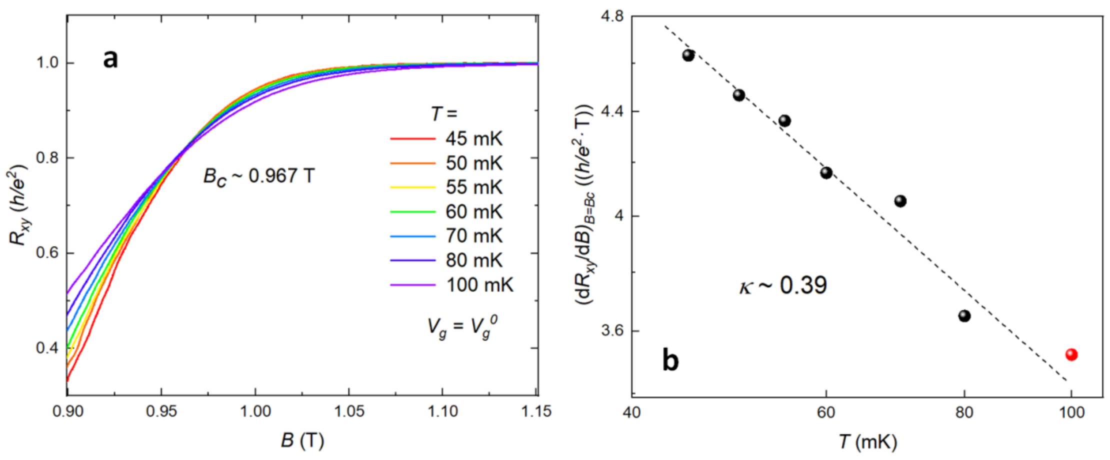

Supplementary Figure 3| Scaling analysis based on R_{xy}. (a) For R_{xy} in the temperature range 45 mK ≤ T ≤ 100 mK, all curves cross at a point B_c ~ 0.967 T. (b) Temperature dependence of $(\frac{dR_{xy}}{dB})_{B=B_c}$ on a log-log scale. The black dashed line is the linear fit.

Supplementary Note 4: Scaling behavior analysis on two more samples

We performed the same measurements on two more samples with the same configuration as the one shown in the main text. In these two samples, the R_{xx} - B curves cross each other only when the temperature is below ~100 mK (**Supplementary Figs. 4a** and **4b**). This observation is the same as that of the sample in the main text. We did the same scaling behavior analysis in the appropriate temperature range and found the critical exponent κ for the QAH to axion insulator transition is ~ 0.37±0.04 and ~ 0.36±0.03, respectively (**Supplementary Figs. 4c** and **4d**). Both values are close to κ ~ 0.38±0.02, which is achieved in the main text.

We pointed out that if we study the scaling behavior based on the data points at higher temperatures when R_{xx} - B curves don't strictly across each other, we will obtain a larger κ, as shown in **Supplementary Figs. 4c** and **4d**. In this temperature regime, the scaling analysis is inappropriate because there is no critical magnetic field and the theoretical assumption for scaling analysis doesn't exist. Therefore, the larger κ value extracted in the higher temperature regime (i.e. 120mK $\leq T \leq$ 300mK) is not reliable. To accurately study the scaling behavior of the QAH to axion insulator phase transition, we should perform our measurements at sufficiently low temperatures, i.e. 45 mK $\leq T <$ 100 mK.

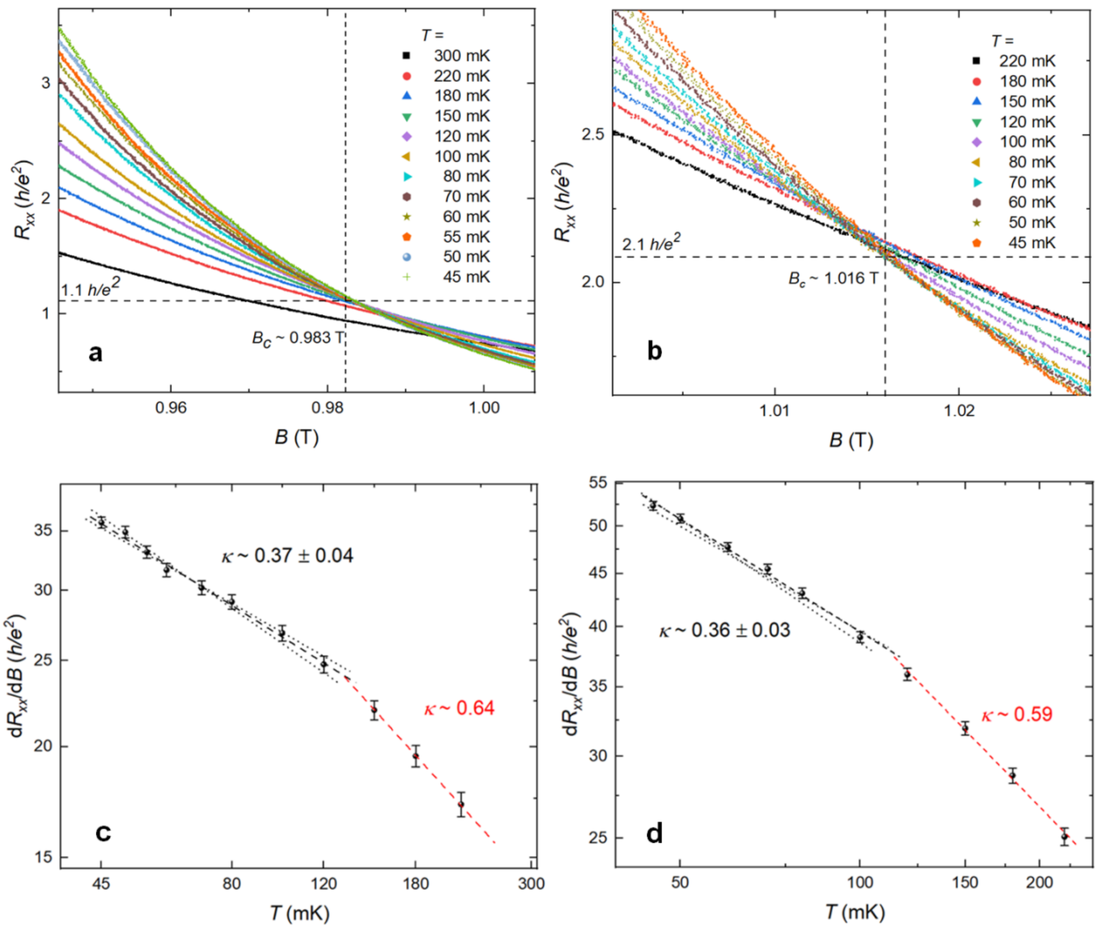

Supplementary Figure 4| Scaling behavior analysis on two more samples. (a, b) R_{xx} vs B curves measured at various temperatures for the second sample (a) and the third sample (b). Both samples have the same configuration as the sample shown in the main text. (c, d) Scaling behavior analysis for the second sample (c) and the third sample (d). The uncertainty in (c) and (d) is estimated from the difference of the maximum slope and minimum slope between the highest temperature point and the lowest temperature point. At the temperature regime where the R_{xx}-B curves don't cross each other at a fixed magnetic field, a larger κ is derived.

Supplementary Note 5: More transports data in the axion insulator regime

We have measured the two-terminal resistance R_{12} at a series of magnetic fields, as shown in **Supplementary Fig. 5**. At B = 0.40 T, the sample shows the most insulating behavior. This is the reason why in the main text, we did the analysis based on the data measured at B = 0.40 T. Moreover, we noted that R_{12} shows similar two-slope behaviors below and above 95 mK under the magnetic field from 0.29 T to 0.60 T.

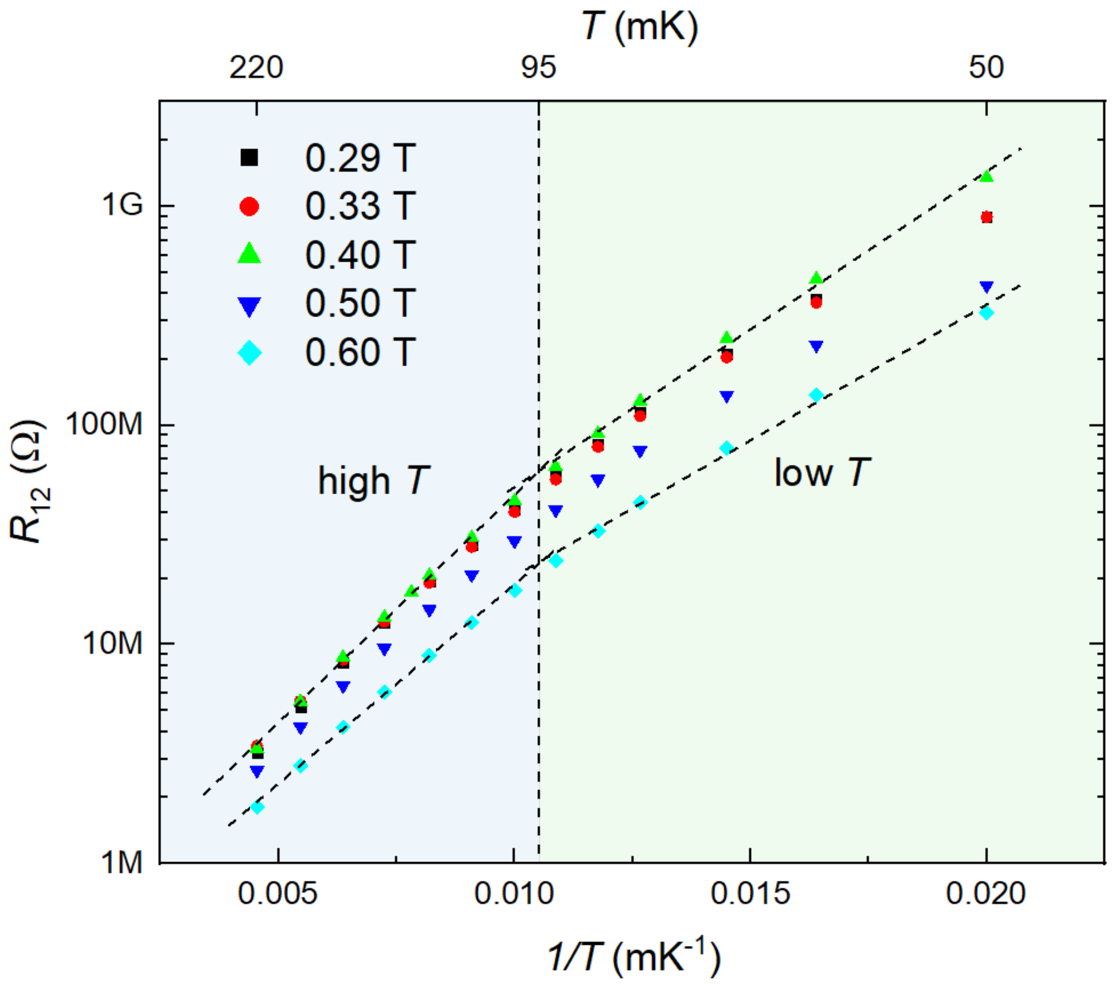

Supplementary Figure 5| R_{12} as a function of temperature at different magnetic fields. The two-terminal DC resistance R_{12} as a function of $1/T$ (bottom) or T (top) at magnetic fields B = 0.29 T, 0.33 T, 0.40 T, 0.50 T, and 0.60 T. The vertical axis is in log scale.

Supplementary Note 6: Optical image of the sample used in the main text

Supplementary Fig. 6 shows the optical image of the Hall bar sample we used in the main text. The effective length *L* is ~ 991 μm and the effective width *W* is ~ 465 μm, so the aspect ratio *L/W* of the Hall bar in our experiment is ~ 2.13.

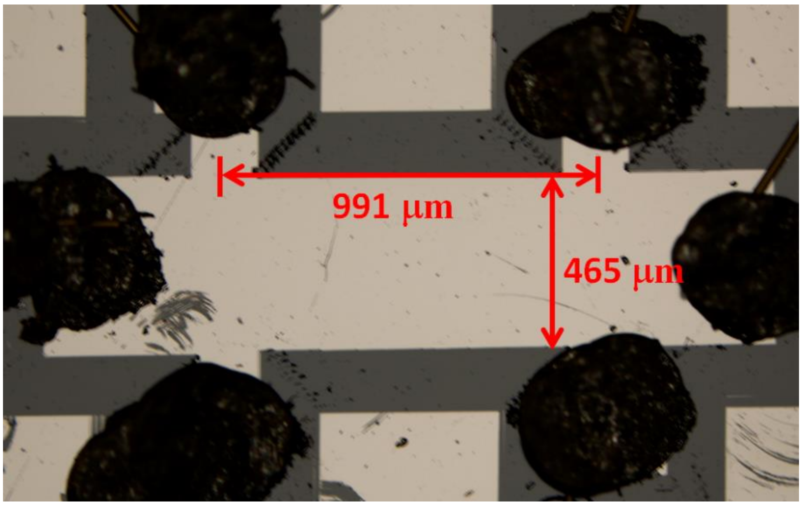

Supplementary Figure 6| The optical image of the sample used in the main text.

Supplementary References

1. Wang P. J., Huang K., Sun J., Hu J. J., Fu H. L., Lin X. Piezo-driven sample rotation system with ultra-low electron temperature. *Rev. Sci. Instrum.* **90**, 023905 (2019).

2. Fu H. L., Wang P. J., Shan P. J., Xiong L., Pfeiffer L. N., West K., Kastner M. A., Lin X. Competing ν=5/2 fractional quantum Hall states in confined geometry. *Proc. Natl. Acad. Sci. U.S.A.* **113**, 12386-12390 (2016).
